# Increase in the OCT angiographic peripapillary vessel density by ROCK inhibitor ripasudil instillation: a comparison with brimonidine

**DOI:** 10.1007/s00417-018-3945-5

**Published:** 2018-03-08

**Authors:** Etsuo Chihara, Galina Dimitrova, Tomoyuki Chihara

**Affiliations:** 1 Sensho-kai Eye Institute, Minamiyama 50-1, Iseda, Uji, Kyoto 611-0043 Japan; 2 Department of Ophthalmology, City General Hospital 8th September, Skopje, Macedonia; 3 Department of Ophthalmology, Kansai Medical University, Hirakata, Osaka, Japan

**Keywords:** OCT angiography, Glaucoma, Radial peripapillary capillary, ROCK inhibitor, Ripasudil, Brimonidine

## Abstract

**Purpose:**

To assess the responses of the superficial peripapillary retinal vessel density (VD) and prelaminar flow index (PLFI) to topical Rho-assisted coiled-coil forming protein kinase (ROCK) inhibitor ripasudil and alpha-2 agonist brimonidine using optical coherence tomography angiography.

**Methods:**

This is a prospective, non-randomized, comparative cohort study. We studied the response of optical coherence tomography angiography (OCTA) parameters to drugs in 24 eyes treated with ripasudil and 23 eyes treated with brimonidine at the Sensho-kai Eye Institute. After division by the signal strength (SS), we compared the responses of peripapillary VD/SS and PLFI/unit area (UA)/SS to topical eye drops in eyes with primary open-angle glaucoma (POAG) and ocular hypertension (OH).

**Results:**

In the superficial peripapillary retina, VD/SS increased significantly in the ripasudil-treated eyes (12.5 ± 21.7%, *P* = 0.018), but not in the brimonidine-treated eyes (− 2.0 ± 13.8%, *P* = 0.484). In the deeper area of the optic disc, the changes in the PLFI/UA/SS in the brimonidine-treated eyes (+ 0.9 ± 8.9%, *P* = 1.00) and ripasudil-treated eyes (− 1.3 ± 8.5%, *P* = 0.241) were not significant. Multivariate discriminant analysis showed that the change in the peripapillary VD/SS was the most important parameter (*P* = 0.0186) for differentiating ripasudil- and brimonidine-treated eyes.

**Conclusions:**

The topical ROCK inhibitor ripasudil enhanced the peripapillary VD in POAG and OH, whereas the alpha-2 agonist brimonidine did not. The PLFI did not respond to either drug.

**Electronic supplementary material:**

The online version of this article (10.1007/s00417-018-3945-5) contains supplementary material, which is available to authorized users.

## Introduction

Vascular insufficiency is one of the main features of glaucomatous optic neuropathy, and fluctuation of the optic disc blood flow is considered a causative factor of nerve loss [1]. Abnormal microcirculation can occur in both the optic nerve head (ONH) and the superficial peripapillary retinal vasculature [2, 3]. The results of recent optical coherence tomography angiography (OCTA) studies have confirmed reduced blood flow in glaucomatous ONH [4], a finding reported previously using the laser speckle technique and microsphere method [5]. However, susceptibility to glaucomatous damage may differ based on the location of vessels. The results of OCTA studies have suggested that superficial vessels such as the radial peripapillary capillaries are susceptible to glaucomatous damage [6], while the microcirculation in the prelaminar area might be resistant to glaucomatous insults in the earliest stage of glaucoma [7].

Among the many parameters that affect blood flow in the ONH, topical antiglaucoma medications may improve microcirculation in the optic nerve head.

The Rho-assisted coiled-coil forming protein kinase (ROCK) inhibitor ripasudil (Glanatec^®^, Kowa Co., Ltd., Aichi, Japan) and its analogues have the potential to reduce intraocular pressure (IOP) in clinical practice [8, 9] and vasodilate vessels [10, 11] and may improve microcirculation in the optic disc [12, 13]. Other than the hypotensive effects, antiglaucoma drugs may suppress oxidative stress [14] and inhibit apoptosis [15]. However, concentration and effects of drugs in and around the ONH after topical instillation have been unknown. If vasodilation of the ONH vessel, which was not proven in the case of brimonidine [16], was confirmed after topical instillation of ripasudil, that would provide pharmacologic evidence that a sufficiently high drug concentration reached the ONH.

In the current prospective study, we compared the effects of ripasudil and brimonidine on the peripapillary vessel density (VD) and prelaminar blood flow index (PLFI) in patients with glaucoma.

## Patients and methods

This was a prospective, non-randomized, comparative study conducted from September 2015 to March 2017 that included 55 eyes of 55 consecutive patients aged 20 years and older. The patients had poorly controlled intraocular pressure (IOP) or visual field progression, required antiglaucoma medication (ripasudil or brimonidine), and were treated at the outpatient service of the Sensho-kai Eye Institute.

The inclusion criteria included the presence of primary open-angle glaucoma (POAG), preperimetric glaucoma, or ocular hypertension (OH). These patients had not undergone previous retinal surgery or laser photocoagulation. Patients who underwent uneventful cataract surgery or glaucoma surgery more than 12 months prior to enrollment into this study were allowed. Previous use of a topical prostaglandin and carbonic anhydrase was allowed; however, patients who used beta blockers were excluded.

The exclusion criteria included the presence of cloudy media, poor-quality OCTA images characterized by doubling of vessel images and artifact lines in the target area, signal strength (SS) below 40, high pretreatment IOP exceeding 30 mmHg, visual acuity (VA) less than 20/40, and mean deviation (MD) below − 20 dB. If vitreous floaters hampered analysis of peripapillary or ONH images, the eyes were excluded from the study. Other exclusion criteria included glaucoma surgery during the study period, a highly deformed optic disc, extensive myopic peripapillary chorioretinal atrophy, iridocyclitis, congenital anomaly or neuro-ophthalmologic diseases, epiretinal membrane, amblyopia, and/or retinal vascular diseases. Patients were also excluded if they had a renal disease, systemic hypertension exceeding 160/90 mmHg, diabetes mellitus, or heart disease, who required a vasodilatory drug such as nitroglycerin, and those who drank alcohol or coffee within 3 h before the examination.

POAG was diagnosed based on a gonioscopic open angle, signs of glaucomatous optic neuropathy using ophthalmoscopy and OCT, and the absence of neuro-ophthalmologic diseases that cause optic nerve atrophy. Glaucomatous visual field defects were studied using the Humphrey Field Analyzer (HFA) (750i, Carl Zeiss Meditec, Tokyo, Japan). Preperimetric glaucoma was defined by positive signs of glaucomatous optic neuropathy on the OCT or nerve fiber layer studies without any visual field defects detected by the HFA (normal glaucoma hemifield test and pattern standard deviation less than 95%). OH was defined by the presence of an open angle and a history of at least two repeated IOP values exceeding 21 mmHg but no glaucomatous visual field defects or glaucomatous optic neuropathy.

We recorded the patients’ age, spherical equivalent refractive error, best-corrected VA, gonioscopy, slit-lamp microscopy, Goldmann applanation tonometric IOP, and the MD and pattern standard deviation of the Humphrey 24-2 visual field test. The presence of systemic hypertension (140/90 mmHg by the Japanese Society of Hypertension Guideline 2014), diabetes mellitus (fasting blood sugar > 126 mg/dl, hemoglobin A1C > 6.5%, Japan Diabetes Society Guideline 2012), and cardiac diseases (angina pectoris, myocardial infarction, arrhythmia) was checked by careful history taking. Twenty-four normal subjects had normal visual fields detected by the HFA.

### Allocation of drugs

Drugs were prescribed in the following order: ripasudil, brimonidine, and then ripasudil.

### Masking

Four experienced technicians who did not know the clinical background of the patients obtained images and uploaded them to the filing system of the Sensho-kai Eye Institute. One investigator (T.C.) who was independent of the examiners recorded numerical data, and the other independent investigator (E.C.) analyzed the numerical data.

When both eyes of a patient were examined, the eyes with better-quality images were enrolled in the study. If both eyes were eligible, the right eye was selected.

The intravisit reproducibility of data was assessed by repeating examinations five times in each of five normal subjects who had no history of intraocular surgery, diabetes mellitus, systemic hypertension, or glaucomatous optic neuropathy and had an IOP below 21 mmHg.

### OCT study

Patients with glaucoma and OH were examined twice before eye drops were prescribed and two times between 1 and 2 months after the initiation of eye drop treatment; the mean data were analyzed. If the OCTA images obtained during one of the two examinations were poor, a third examination was performed. The compliance of patients with eye drop instillation was confirmed by patient inquiry. The methodology of obtaining OCTA images was previously reported [7]. Briefly, the enrolled eyes were studied using spectral-domain OCT equipped with an angiographic function (2014.2.0.90 software version) (AngioVue, RTVue XR OCT, Optovue, Fremont, CA, USA), and 4.5 × 4.5-mm images were obtained. The parameters studied were the global loss volume (GLV) of the ganglion cell complex, circumpapillary retinal nerve fiber layer thickness (cpNFLT), signal strength (SS), vessel density (VD) by measuring 0 to 80 μm from the internal limiting membrane within 700 μm of the disc margin, and prelaminar vascular flow index (PLFI). To study the PLFI, Elschnig’s scleral ring was marked, the vessels in the cylindrical column 50 to 250 μm from the surface, which included the prelaminar area, were highlighted, and the mean decorrelation was measured as the flow index [17]. To avoid the effect of disc size, the PLFI was divided by the area measured (in square millimeters), i.e., the PLFI/unit area (UA) was used for the analysis.

The wavelength of the OCTA device was 840 nm. The definition of the GLV was reported elsewhere [18]. Elimination of projection artifacts was based on the slab subtraction algorithm [19].

The VD was defined as the percentage of signal-positive pixels/area of interest. The algorithm to detect the disc margin was reported previously [20]. Considering patient-specific media opacity, tissue vascularity, refractive error, and waning of the SS, the VD/SS and PLFI/UA/SS were also evaluated.

The mean perfusion pressure (PP) was defined by the formula of Yaoeda et al., where PP = 2/3(diastolic blood pressure [BP] + 1/3(systolic BP − diastolic BP) − IOP) [21].

The institutional review board of the Sensho-kai Eye Institute approved the study design. All subjects provided informed consent after they received an explanation of the study nature and possible consequences. The study adhered to the tenets of the Declaration of Helsinki.

### Statistical analysis

The Bell Curve Excel Tokei 2016 (SSRI, Tokyo, Japan) was used to assess data by Wilcoxon signed-rank test, Mann-Whitney *U* test, chi-squared test, Tukey’s multiple comparison, Pearson’s correlation coefficient, and multivariate discriminant analysis.

The sample size was calculated based on the effective size and power of the ΔVD/SS. The power to calculate the difference between the brimonidine and the ripasudil cohorts to the level of 5% significance was 0.95 for two-sided data analysis. The appropriate sample size to obtain a significance level of 0.05 in two-sided paired data was 23.

## Results

One eye of 55 patients (27 assigned to brimonidine and 28 to ripasudil) and one eye of 24 normal subjects were enrolled in this study; eight patients were excluded after enrollment (one patient because of poor compliance, three because of diabetes mellitus diagnosed later, three with poor-quality images, and one with high systemic BP during the follow-up periods). Ultimately, 24 eyes of 24 patients assigned to ripasudil and 23 eyes of 23 patients assigned to brimonidine were included. Thirty-six eyes had POAG (including two with preperimetric glaucoma), and 11 eyes had OH.

Nine eyes had new prescriptions, and 15 eyes had added-on prescriptions of ripasudil, respectively; the numbers for brimonidine were 14 and nine eyes, respectively, (*P* = 0.11, by the chi-squared test).

In five normal subjects, the intravisit reproducibility of the data for the SS, VD, PLFI, VD/SS, PLFI/UA, and PLFI/UA/SS was assessed by repeating examinations five times. The mean and standard deviation of the coefficient of variation of each parameter were 4.3 ± 1.6, 3.4 ± 3.1, 6.3 ± 2.7, 2.7 ± 1.6, 6.6 ± 3.9, and 7.6 ± 3.7%, respectively.

Table 1 shows the demographic data of the participants. There were no significant differences in age, IOP, best-corrected visual acuity, spherical equivalent refractive error, MD, GLV, cpNFLT, VD, or PLFI/UA (*P* = 0.426, 0.774, 0.367, 0.855, 0.140, 0.690, 0.774, 0.617, and 0.383, respectively) between the brimonidine and the ripasudil cohorts by Tukey’s multiple comparison.

**Table 1:** Baseline characteristics of 47 participants and 24 control subjects (mean ± standard deviation)

	Age	IOP	BCVA (logMAR)	SQRE	MD	GLV	cpNFLT	VD	PLFI/UA
Brimonidine *n* = 23	63.3 ± 17.8	20.4 ± 4.6	− 0.029 ± 0.165	− 1.74 ± ±6.85	− 7.01 ± 7.84	17.21 ± 10.6	76.5 ± 14.2	34.7 ± 8.7	0.585 ± 0.115
Ripasudil *n* = 24	68.0 ± 9.4	21.1 ± 2.9	− 0.079 ± 0.101	− 2.65 ± 4.87	− 4.33 ± 4.62	15.36 ± 11.3	79.2 ± 16.1	32.1 ± 10.8	0.626 ± 0.103
Normal *n* = 24	57.4 ± 10.2	16.5 ± 3.0	− 0.130 ± 0.094	− 1.75 ± 2.95	0.100 ± 0.55	2.52 ± 2.5	97.9 ± 8.8	49.0 ± 8.2	0.718 ± 0.099
Brimonidine vs. ripasudil P*	0.426	0.774	0.367	0.855	0.140	0.690	0.774	0.617	0.383
Brimonidine vs. normal P*	0.276	0.001	0.018	0.999	< 0.001	< 0.001	< 0.001	< 0.001	< 0.001
Ripasudil vs. normal P*	0.017	< 0.001	0.311	0.838	0.012	< 0.001	< 0.001	< 0.001	0.009

*IOP* intraocular pressure, *BCVA* best-corrected visual acuity, *SQRE* spherical equivalent refractive error, *MD* mean deviation, *GLV* global loss volume, *cpNFLT* circumpapillary retinal nerve fiber layer thickness, *VD* vessel density, *PLFI/UA* prelaminar flow index/unit area, *logMAR* logarithm of the minimum angle of resolution, P* P value by Tukey’s multiple comparison

Table 2 shows the changes in the IOP and perfusion pressure by eye drop instillation. The IOP decreased significantly by 12.1 ± 14.8% (*P <* 0.001) in the ripasudil-treated eyes and 21.8 ± 12.6% (*P* < 0.001 Wilcoxon signed-rank test) in the brimonidine-treated eyes. The IOP after brimonidine treatment was significantly (*P* = 0.0103, Mann-Whitney *U* test) lower than in the ripasudil-treated eyes (Table 2). The baseline PP values in the ripasudil-and brimonidine-treated cohorts were 51.7 ± 10.0 and 48.6 ± 13.3 mmHg, respectively. The post-treatment PP values of 52.8 ± 11.1 mmHg in the ripasudil-treated cohort did not differ significantly from that of the baseline (*P* = 0.100); however, the post-treatment PP of 51.3 ± 7.9 mmHg in the brimonidine-treated cohort was significantly higher than that of baseline (*P* = 0.0023 Wilcoxon signed-rank test). Changes in GLV or cpNFLT by brimonidine or ripasudil treatment were not significant (*P* = 0.211–0.879; appendix Table 4).

**Table 2:** Changes in studied parameters by topical eye drops

	Baseline IOP	Post-treatment IOP	P*	% change	Baseline PP	Post-treatment PP	P*	% change
Brimonidine *n* = 23	20.4 ± 4.6	15.7 ± 3.3	< 0.001**	− 21.8 ± 12.6	48.6 ± 13.3	51.3 ± 7.9	0.0023**	15.2 + 28.8
Ripasudil *n* = 24	21.1 ± 2.9	18.3 ± 3.0	< 0.001**	− 12.1 ± 14.8	51.7 ± 10.0	52.8 ± 11.1	0.1005	3.7 ± 16.5
*P*	0.983	0.0103*		0.0086**	0.315	0.615		0.14

P* = *P* value (Wilcoxon signed-rank test) for difference between baseline and post-treatment. P = *P* value (Mann-Whitney *U* test) for comparison between brimonidine- and ripasudil-treated cohorts

*IOP* intraocular pressure (mmHg), *PP* perfusion pressure (mmHg)

*Significant with *P* < 0.05; **Significant with *P* < 0.01

**Fig. 1:**
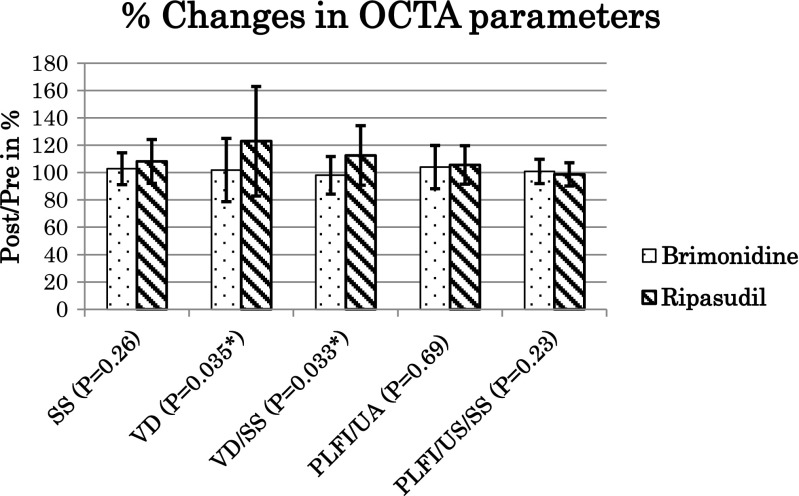
Changes in optical coherence tomography angiography parameters by brimonidine (dotted columns) and ripasudil (striped column) treatment. The increases in the vessel density (VD) and VD/signal strength (SS) in the ripasudil-treated cohort were significant by 22.9 ± 40% (*P* = 0.025) and 12.5 ± 21.5% (*P* = 0.018 by Wilcoxon signed-rank test), respectively, while those in the brimonidine-treated eyes of 1.8 ± 23.2% (*P* = 0.903) and − 2.0 ± 13.8% (*P* = 0.484 by Wilcoxon signed-rank test), respectively, were not significant (Table 3). There were significant differences in the percentage increase in the VD and VD/SS between ripasudil- and brimonidine-treated cohorts (*P* = 0.035 and *P* = 0.033, respectively) by the Mann-Whitney *U* test. On the other hand, the SS, prelaminar flow index/unit area (PLFI/UA) and the PLFI/UA/SS did not change after brimonidine or ripasudil treatment, and there was no difference between ripasudil- and brimonidine-treated cohorts by *P* = 0.26, *P* = 0.69, and 0.23, respectively (*P* values, by Mann-Whitney *U* test).

The responses of the OCTA parameters to brimonidine and ripasudil are shown in Table 3. Increases in the peripapillary VD (22.9 ± 40.0%, *P* = 0.025) and VD/SS (12.5 ± 21.7%, *P* = 0.018) by ripasudil were significant by Wilcoxon signed-rank test; however, changes in these parameters in the brimonidine-treated cohort (1.8 ± 23.2%, *P* = 0.903 and − 2.0 ± 13.8% *P* = 0.484) were not significant. Changes in SS, PLFI/UA, and PLFI/UA/SS in brimonidine-treated eyes (2.8 ± 11.7%, *P* = 0.162; 4.0 ± 15.8%, *P* = 0.248; 0.9 ± 8.9%, *P* = 1.0, respectively) and in ripasudil-treated eyes (8.1 ± 16.0%, *P* = 0.098; 5.6 ± 14.0%, *P* = 0.241; and −1.3 ± 8.5%, P = 0.241, respectively) were not significant (Table 3). When the percentage change of the OCTA parameters was compared between brimonidine-and ripasudil-treated cohorts, only the differences in VD (*P* = 0.035) and VD/SS (*P* = 0.033) were significant by Mann-Whitney *U* test (Fig. 1).

**Table 3:** Responses of optical coherence tomography angiography parameters to brimonidine and ripasudil treatments

	Baseline	Post-treatment	% changes	*P*
Signal strength (SS)				
Brimonidine	56.0 ± 9.2	57.1 ± 7.9	102.8 ± 11.7	0.162
Ripasudil	55.4 ± 8.7	59.1 ± 7.0	108.1 ± 16.0	0.098
Vessel density (VD)				
Brimonidine	34.7 ± 8.7	34.8 ± 9.2	101.8 ± 23.2	0.903
Ripasudil	32.1 ± 10.8	36.7 ± 8.7	122.9 ± 40.0	0.025*
VD/SS				
Brimonidine	0.614 ± 0.083	0.602 ± 0.112	98.0 ± 13.8	0.484
Ripasudil	0.568 ± 0.125	0.620 ± 0.093	112.5 ± 21.7	0.018*
Prelaminar flow index (PLFI)/unit area (UA)				
Brimonidine	0.585 ± 0.115	0.603 ± 0.125	104.0 ± 15.8	0.248
Ripasudil	0.626 ± 0.103	0.650 ± 0.064	105.6 ± 14.0	0.241
PLFI/UA/SS				
Brimonidine	0.0105 ± 0.0017	0.0106 ± 0.0017	100.9 ± 8.9	1
Ripasudil	0.0113 ± 0.0012	0.0111 ± 0.0010	98.7 ± 8.5	0.241

*P*: by Wilcoxon signed-rank test to compare between baseline and post-treatment data

*VD/SS* vessel density/signal strength, *PLFI/UA* prelaminar flow index/unit area, *PLFI/UA/SS* prelaminar flow index/unit area/signal strength

*Significant *P* < 0.05

**Fig. 2:**
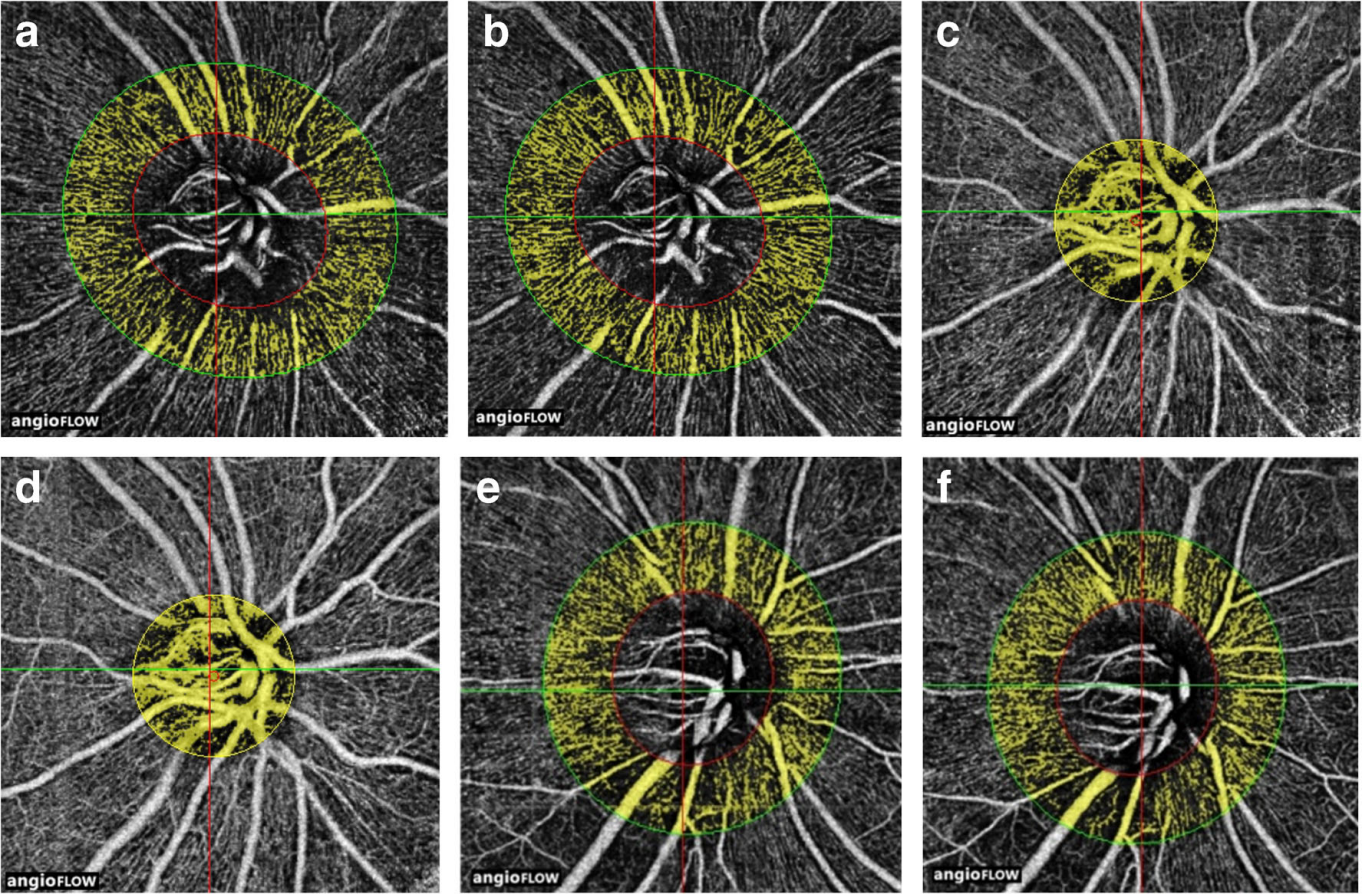
Increased peripapillary vessel density (VD) after ripasudil treatment (**b**) in comparison to baseline VD (**a**). This finding is in contrast to stationary vasculature at the prelaminar area in the same eye (**c** baseline and **d** after treatment) and stationary peripapillary vessel density after brimonidine treatment (**e** baseline and **f** after treatment). **a** Baseline superficial peripapillary vessels before ripasudil treatment. The vessel density/signal strength (VD/SS) was 0.741 (VD = 43; SS = 58). The highlighted area is 700 μm from the disc margin. **b** Peripapillary VD after ripasudil treatment. Peripapillary VD and VD/SS increased from 43 and 0.741 to 50 and 0.820 (increase of 16.2 and 10.7%, respectively) after start of ripasudil treatment. This increase was significant (Fig. 1 and Table 3). SS was 61 for this image. **c** Baseline prelaminar area vessels (50–250 μm from the surface) examined at the same time as **a** acquisition. The PLFI/UA and PLFI/UA/SS before ripasudil treatment were 0.630 and 0.0109, respectively (PLFI = 1.242, area examined = 1.97 mm^2^, SS = 58). **d** Stationary prelaminar vessels (deeper vessels of **b**) after ripasudil treatment started. In contrast to the superficial peripapillary vessels, the post-treatment PLFI/UA (0.670) and PLFI/UA/SS (0.0110) (PLFI = 1.333, area examined = 1.99 mm^2^, SS = 61) did not change much from the baseline of 0.630 and 0.0109, which was increased + 6.3 and + 0.9%, respectively. When percentile changes in PLFI/UA and PLFI/UA/SS were compared between ripasudil- and brimonidine-treated cohorts, differences were not significant (*P* = 0.69 and 0.23, respectively, by Mann-Whitney *U* test: Fig. 1 and Table 5 in appendix). **e** Baseline peripapillary superficial vessels before brimonidine treatment started. **f** Stationary peripapillary vessel density after topical instillation of brimonidine started. The VD/signal strength of 0.690 after brimonidine treatment started did not change markedly from the pretreatment level of 0.700

Figure 2 shows a representative case, who shows increased VD/SS despite stationary PLFI/UA after ripasudil treatment (Fig. 2a–d) and stationary VD/SS and PLFI/UA after brimonidine treatment (Fig. 2e, g). Horizontal section images of Fig. 2c, d are shown in the appendix (Figs. 2g and 2h).

The Pearson’s correlation coefficients between ΔVD/SS and ΔPP and ΔIOP were *r* = − 0.304 (*P* = 0.251) and *r* = − 0.205 (*P* = 0.446), respectively, for the brimonidine cohort, *r* = − 0.340 (*P* = 0.134) and *r* = 0.205 (*P* = 0.386), respectively, for the ripasudil cohort, and were not significant in this study.

**Fig. 3:**
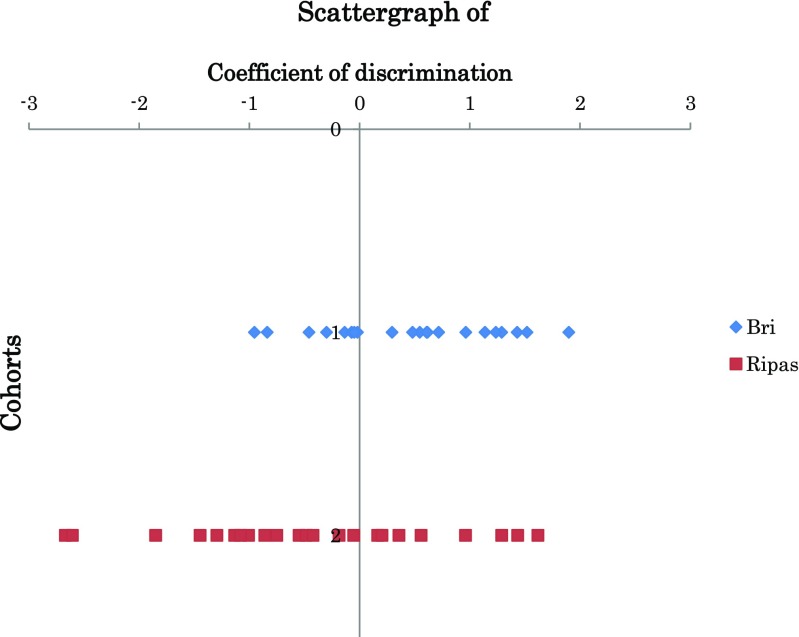
Scatter graph of the discriminant coefficient *A* (*A* = 9.39ΔVD/SS + 0.183ΔIOP − 0.528). Multivariate analysis showed that the change in ΔVD/SS (*P* = 0.0186) is the most important parameter of the coefficient for discrimination between brimonidine- and ripasudil-treated cohorts. The center of the coefficient for brimonidine-treated eyes was 0.4464 and that of ripasudil was − 0.4270. Bri brimonidine, Ripas ripasudil. Dots are discriminant coefficient (*A*) of each patient

The associations between the post- and pretreatment differences in IOP, VD/SS, PLFI/UA/SS, GLV, and cpNFLT and the drugs were analyzed by multivariate analysis (discriminant analysis with a method of increasing and decreasing variables). When the ΔIOP, ΔVD/SS, ΔPLFI/UA/SS, ΔGLV, and ΔcpNFLT were independent parameters and the use of ripasudil and brimonidine was set as a dependent parameter, the most significant parameter that differentiated ripasudil- and brimonidine-treated eyes was ΔVD/SS (*P* = 0.0186) followed by ΔIOP (*P* = 0.099), ΔcpNFLT (*P* = 0.249), ΔGLV (*P* = 0.384), and ΔPLFI/UA/SS (*P* = 0.454). Because the significantly effective parameters for calculating the coefficient of discrimination were selected at the *P* = 0.2 level (Bell Curve Tokei Manual), the obtained discriminant equation for calculating discriminative coefficient A was *A* = 9.39ΔVD/SS + 0.183ΔIOP − 0.528, and its discriminative power was 59.1% for brimonidine and 65.2% for ripasudil. The centers of the discriminative coefficient for brimonidine and ripasudil were 0.4464 and − 0.4270, respectively (Fig. 3).

## Discussion

Many studies have been published concerning improved microcirculation in the ONH resulting from topical antiglaucoma medications. Other than the direct effects of the drugs, the reduced IOP itself [22] and the systemic BP or PP might affect the optic disc or retinal perfusion [23, 24]. The combination of high IOP and low systolic BP led to dysregulation of the optic disc flow in rhesus monkeys [25]. In the current study, associations between ΔVD/SS and ΔPP and ΔIOP were not significant in the ripasudil-treated patients (*P* = 0.134 and *P* = 0.386, respectively). The reduction in the IOP was more pronounced in the brimonidine-treated cohort (21.8%) than in the ripasudil-treated cohort (12.1%), and the increase in the PP was significant only in the brimonidine-treated cohort (*P* = 0.0023 Table 2). Despite a theoretically favored hemodynamic situation in the brimonidine-treated eyes, an increase in the peripapillary VD was noted only in the ripasudil cohort. Therefore, the effects of the PP and IOP on optic disc microcirculation may be less than the pharmacological effects of ripasudil.

ROCK inhibitors disrupt actin filaments and inhibit contraction of the smooth muscle [26, 27]. Fasudil, an analogue of ripasudil, has a potent vasodilatory effect and is clinically used to treat arteriosclerosis, vasospasm, and hypertension [10, 11]. The effect of ripasudil is 5 to 10 times that of fasudil [14, 28], and an intravitreal injection of ripasudil increased the retinal blood flow in experimental studies [29]. Thus, if the concentration of ripasudil is sufficiently high, vasodilation might occur in the ONH. Some reports have suggested increased blood flow in the ONH resulting from topical instillation of a ROCK inhibitor [12, 13].

Interestingly, the effect of ripasudil was found in the OCT angiographic superficial peripapillary area and not in the prelaminar area. If penetration of ripasudil occurs via the periocular transscleral and/or uveoscleral routes [30], the ripasudil concentration might be higher in the peripapillary retina than in the ONH and cause vasodilation of only the peripapillary vessels [31]. The presence of Kuhnt intermediary tissue as a barrier between the choroid and the ONH might hamper drug diffusion from the choroid or sclera to the ONH [32]. Different responses in the peripapillary vessels and prelaminar area vessels to the drugs, IOP, or glaucomatous insults might be another reason [33, 34].

The current results suggest that ripasudil reaches the ONH in a sufficiently high concentration to vasodilate the peripapillary vessels. However, the association between vasodilation and oxidative stress or neuroprotection is unknown. Clinical evidence that supports neuroprotection of the optic nerve has not yet been reported for ripasudil and is a subject for future studies.

Improvement of the blood flow by brimonidine was not confirmed in this study or in a previous study [16]. Therefore, the neuroprotective effect of brimonidine, if it exists [35], might not be attributed to changes in the microcirculation but to other mechanisms such as modification of Bcl-2 [15].

The flow index in the current study was defined as an average decorrelation at the segmented area [20]. The correlation between the decorrelation and the true blood flow is not parallel but is a sigmoid association [36]; thus, the flow index should be considered one of the indicators of blood flow. Because noise below the level of 0.125 was cut and the blood flow signal from the large vessels was saturated, the changes in the flow index in the current study are expected to reflect the capillary level blood flow changes. In our previous study, the flow index was correlated closely with the VD (Pearson’s correlation coefficient of 0.93 to 0.99 [24]) and might be considered as a parameter that is nearly identical to the VD.

### Study limitations

In this study, segmentation of the prelaminar and laminar tissue is poor, and the PLFI covers a large part of the prelaminar area and a small part of the lamina cribrosa. However, the poor segmentation between the lamina and the prelaminar region may not affect the result much, because in an experimental setting, the blood flow at the laminar tissue did not differ significantly from that in the prelaminar area [37]. For future studies, precise segmentation of the lamina cribrosa is desirable to elaborate the pathophysiology at the prelaminar area.

When the OCT angiographic quantitative data are analyzed, care must be taken regarding the limits in the ability of OCT angiography to assess blood flow and modification factors. Current OCT angiography technology is still immature to assess the blood flow. This may be the reason why the OCT angiographic blood flow parameters are not validated well and need further improvement. The type of software, algorithm to reduce projection artifacts and machine used, severity of glaucoma, refractive error, age, and retinal thickness may affect the VD [7]. Other than the parameters studied in this report, diabetes mellitus, renal function, systemic drugs, and others might affect the results and are subjects for future studies.

In conclusion, topical ripasudil enhanced the VD of the peripapillary superficial retina but not the PLFI. This finding differed from the response in cases treated with topical brimonidine, in which both the peripapillary and the prelaminar area microcirculation remained unchanged.

## Electronic supplementary material

Fig. 2g (GIF 4kb)

High resolution image (TIFF 1324kb)

Fig. 2h (GIF 4kb)

High resolution image (TIFF 1189kb)

ESM 1 (XLSX 69kb)

Appendix Table 4 (DOCX 15kb)

Appendix Table 5 (DOCX 17kb)
