# Supplementary figures and images for: Increase in the OCT angiographic peripapillary vessel density by ROCK inhibitor ripasudil instillation: a comparison with brimonidine
Source: Graefes Arch Clin Exp Ophthalmol. 2018 Mar 8;256(7):1257–64. doi: 10.1007/s00417-018-3945-5 (PMC6006239; doi:10.1007/s00417-018-3945-5)

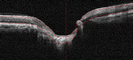

Supplement: Supplementary file 1 — (GIF 4kb) [file 417_2018_3945_Fig4_ESM.gif]

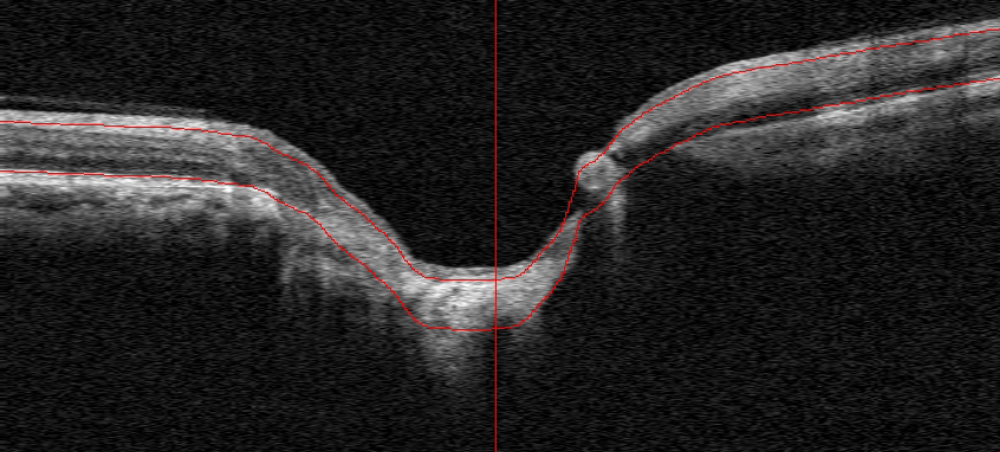

Supplement: Supplementary file 2 — High resolution image (TIFF 1324kb) [file 417_2018_3945_MOESM1_ESM.tif]

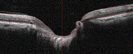

Supplement: Supplementary file 3 — (GIF 4kb) [file 417_2018_3945_Fig5_ESM.gif]

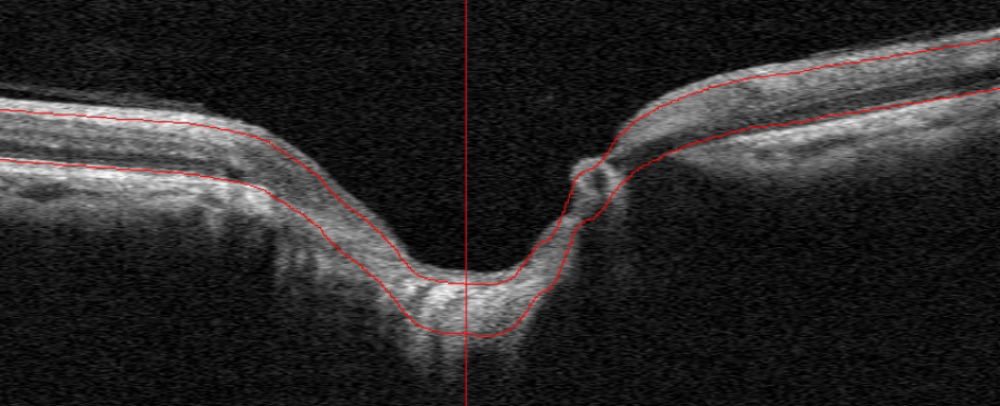

Supplement: Supplementary file 4 — High resolution image (TIFF 1189kb) [file 417_2018_3945_MOESM2_ESM.tif]
