# Supplementary material for: Increase in the OCT angiographic peripapillary vessel density by ROCK inhibitor ripasudil instillation: a comparison with brimonidine
Source: Graefes Arch Clin Exp Ophthalmol. 2018 Mar 8;256(7):1257–64. doi: 10.1007/s00417-018-3945-5 (PMC6006239; doi:10.1007/s00417-018-3945-5)
Supplement: Supplementary file 6 — (DOCX 15kb) [file 417_2018_3945_MOESM4_ESM.docx]

Table 4. Changes in GLV and cpNFLT by drug treatment

|  | GLV | | | cpNFLT | | |
| --- | --- | --- | --- | --- | --- | --- |
|  | Baseline | post-treatment | P* by Wilcoxon signed-rank test | pretreatment | post-treatment | P* by Wilcoxon signed-rank test |
| Brimonidine | 17.2±10.6 | 16.5±10.5 | 0.249 | 76.5±14.2 | 77.6±13.2 | 0.211 |
| Ripasudil | 15.4±11.3 | 15.2±11.3 | 0.511 | 79.2±16.1 | 79.1±15.6 | 0.879 |
| P by Mann-Whitney U test | 0.442 | 0.61 |  | 0.565 | 0.766 |  |

*P** = *P* value (Wilcoxon signed-rank test) for difference between baseline and post-treatment data

P= P value (Mann-Whitney U test) for comparison between brimonidine- and ripasudil-treated cohorts

GLV: global loss volume, cpNFLT: circumpapillary retinal nerve fiber layer thickness
