# Supplementary material for: Increase in the OCT angiographic peripapillary vessel density by ROCK inhibitor ripasudil instillation: a comparison with brimonidine
Source: Graefes Arch Clin Exp Ophthalmol. 2018 Mar 8;256(7):1257–64. doi: 10.1007/s00417-018-3945-5 (PMC6006239; doi:10.1007/s00417-018-3945-5)
Supplement: Supplementary file 7 — (DOCX 17kb) [file 417_2018_3945_MOESM5_ESM.docx]

Appendix

Table 5. Detailed response of optical coherence tomography angiography parameters to brimonidine and ripasudil.

| Signal strength | Drug | Baseline | post-treatment | ΔSS (Post-Pre) | Post/Pre % | P by Wilcoxon signed-rank test |
| --- | --- | --- | --- | --- | --- | --- |
|  | Brimonidine N=23 | 56.0±9.2 | 57.1±7.9 | 1.05±6.57 | 102.8±11.7 | 0.162 |
|  | Ripasudil N=24 | 55.4±8.7 | 59.0±7.0 | 3.5±8.3 | 108.1±16.0 | 0.098 |
|  | P by Mann-Whitney U Test | 0.873 | 0.431 | 0.221 | 0.255 |  |
| VD | Drug | Baseline | post-treatment | ΔSS (Post-Pre) | Post/Pre % | P by Wilcoxon Signed-Rank Test |
|  | Brimonidine N=23 | 34.7±8.7 | 34.8±9.2 | 0.16±7.2 | 101.8±23.2 | 0.903 |
|  | Ripasudil N=24 | 32.1±10.8 | 36.7±8.7 | 4.5±8.7 | 122.9±40.0 | 0.025* |
|  | P by Mann-Whitney's U Test | 0.349 | 0.475 | 0.053 | 0.035* |  |
| VD/SS | Drug | Baseline | post-treatment | ΔSS (Post-Pre) | Post/Pre % | P by Wilcoxon Signed-Rank Test |
|  | Brimonidine N=23 | 0.614±0.083 | 0.602±0.112 | -0.0125±0.082 | 98.0±13.8 | 0.484 |
|  | Ripasudil N=24 | 0.568±0.125 | 0.620±0.093 | 0.052±0.090 | 112.5±21.7 | 0.018* |
|  | P by Mann-Whitney U Test | 0.292 | 0.587 | 0.035* | 0.033* |  |
| PLFI/UA | Drug | Baseline | post-treatment | ΔSS (Post-Pre) | Post/Pre % | P by Wilcoxon Signed-Rank Test |
|  | Brimonidine N=23 | 0.585±0.115 | 0.603±0.125 | 0.019±0.087 | 104.0±15.8 | 0.248 |
|  | Ripasudil N=24 | 0.626±0.103 | 0.650±0.064 | 0.024±0.079 | 105.6±14.0 | 0.241 |
|  | P by Mann-Whitney U Test | 0.202 | 0.317 | 0.848 | 0.686 |  |
| PLFI/UA/SS | Drug | Baseline | post-treatment | ΔSS (Post-Pre) | Post/Pre % | P by Wilcoxon Signed-Rank Test |
|  | Brimonidine N=23 | 0.0105±0.0017 | 0.0106±0.0017 | 4.3E05±8.7E05 | 100.9±8.9 | 1 |
|  | Ripasudil N=24 | 0.0113±0.0012 | 0.0111±0.0010 | -2.1E04±9.3E04 | 98.7±8.5 | 0.241 |
|  | P by Mann-Whitney U Test | 0.101 | 0.307 | 0.225 | 0.233 |  |

VD: vessel density, VD/SS: vessel density/signal strength, PLFI/UA: prelaminar flow index/unit area, PLFI/UA/SS: prelaminar flow index/unit area/signal strength
